# A hierarchical study for urban statistical indicators on the prevalence of COVID-19 in Chinese city clusters based on multiple linear regression (MLR) and polynomial best subset regression (PBSR) analysis

**DOI:** 10.1038/s41598-022-05859-8

**Published:** 2022-02-04

**Authors:** Ali Cheshmehzangi, Yujian Li, Haoran Li, Shuyue Zhang, Xiangliang Huang, Xu Chen, Zhaohui Su, Maycon Sedrez, Ayotunde Dawodu

**Affiliations:** 1 Department of Architecture and Built Environment, University of Nottingham Ningbo China, 199 Taikang East Road, PMB 429, Ningbo, 315100 China; 2 Urban Innovation Lab, Ningbo, 315100 Zhejiang China; 3 Network for Education and Research on Peace and Sustainability (NERPS), Hiroshima University, Hiroshima, 739-8530 Japan; 4 UT Health San Antonio, San Antonio, TX 78229 USA

**Keywords:** Environmental social sciences, Diseases

## Abstract

With evidence-based measures, COVID-19 can be effectively controlled by advanced data analysis and prediction. However, while valuable insights are available, there is a shortage of robust and rigorous research on what factors shape COVID-19 transmissions at the city cluster level. Therefore, to bridge the research gap, we adopted a data-driven hierarchical modeling approach to identify the most influential factors in shaping COVID-19 transmissions across different Chinese cities and clusters. The data used in this study are from Chinese officials, and hierarchical modeling conclusions drawn from the analysis are systematic, multifaceted, and comprehensive. To further improve research rigor, the study utilizes SPSS, Python and RStudio to conduct multiple linear regression and polynomial best subset regression (PBSR) analysis for the hierarchical modeling. The regression model utilizes the magnitude of various relative factors in nine Chinese city clusters, including 45 cities at a different level of clusters, to examine these aspects from the city cluster scale, exploring the correlation between various factors of the cities. These initial 12 factors are comprised of ‘Urban population ratio’, ‘Retail sales of consumer goods’, ‘Number of tourists’, ‘Tourism Income’, ‘Ratio of the elderly population (> 60 year old) in this city’, ‘population density’, ‘Mobility scale (move in/inbound) during the spring festival’, ‘Ratio of Population and Health facilities’, ‘Jobless rate (%)’, ‘The straight-line distance from original epicenter Wuhan to this city’, ‘urban per capita GDP’, and ‘the prevalence of the COVID-19’. The study’s results provide rigorously-tested and evidence-based insights on most instrumental factors that shape COVID-19 transmissions across cities and regions in China. Overall, the study findings found that per capita GDP and population mobility rates were the most affected factors in the prevalence of COVID-19 in a city, which could inform health experts and government officials to design and develop evidence-based and effective public health policies that could curb the spread of the COVID-19 pandemic.

## Introduction

### The general process of the paper

#### Background on the COVID-19 pandemic

In December 2019, a pneumonia virus of unknown cause was detected in a wholesale seafood market in Wuhan, China. It was discovered and reported by the China Novel Coronavirus Investigating and Research Team^1^. It then spread widely across the globe and turned into a pandemic event. The World Health Organization (WHO) then declared this public health threat of coronavirus disease as a new Coronavirus SARS-Cov-2. The pandemic was called “COVID-19” on March 11, 2020^2^. Social mobility and economic growth have been limited and slowed down in many regions by self-imposed quarantine and travel restriction policies, widely known as primary restrictive policies. Also, many sectors, including all service industries, are affected severely by the ongoing pandemic^3^. Regional assessments of the impact of the COVID-19 pandemic are becoming increasingly urgent and vital in providing preventive forecasts for policymakers.

#### The novelty of this study

Many articles related to the COVID-19 are only explored at the country or city level, including a large number of influencing factors, such as meteorological factors^4^ and spatial and demographic factors^5^ affecting the incidence of Covid-19 among cities and counties. However, in China, where the first COVID-19 case is found, there is a more prominent way to distinguish between cities, provinces, and urban agglomerations. In this paper, nine different urban agglomerations are selected and divided into three different levels based on their development status. The study investigates the relationship between 12 identified variables and the prevalence of the COVID-19 in urban agglomerations of different levels.

In this paper, two models are used to determine correlations between 12 variables and three different urban agglomeration grades (i.e., high, middle, low) and six city tiers (based on China’s nationwide tier system). The first is a linear regression model, and the second is polynomial Best Subset regression. Multiple linear regression is linear modeling of ordinary least-squares (OLS) regression for multiple explanatory variables to explain the linear relationship of a dependent variable. Since the outbreak of the COVID-19 pandemic, multiple linear regression (MLR) has been widely used in various cases and mortality-related inquiries analysis. However, in this study, multiple linear regression and polynomial best subset regression are combined to explore possible influencing factors of the COVID-19 prevalence in cities and urban agglomerations. Compared to previous studies in the area, this is a new approach, which is also the novelty of this paper. This novelty also feeds into an enhanced methodological package and higher accuracy and validity of results.

#### The context of study

Cities are flows of human interactions weaved by space and systems. To build better cities, we must first gain an in-depth understanding of the interactions between humans, space, and systems—the very nodes, networks, and connections that forge the fabric of cities^6^. The studies should also consider regional networks between cities, towns, and their surrounding built and natural environments. In the fight against the novel coronavirus (COVID-19) in global regionality (urban/regional), a few studies found that these tangible differences included response measures, population index, economy, and infrastructure of cities^7^. These factors indicate apparent differences in the resilience against the outbreak^8–13^. Compared with cities worldwide, China has formed a relatively distinct city classification system, such as province-level cities, prefecture-level cities, and county-level cities^14^. Since 2000, more than 93% of the global urban agglomeration research literature has focused on the research of Chinese urban agglomerations^15^. China’s urban agglomeration theory is a modeling study worthy of reference to research the regional blocks’ induced etiology. Based on the policies of the Chinese central government, the three major coastal city clusters act as the exemplary role of China’s urban clusters, followed by supporting the development of central urban clusters, and finally supporting the development of urban clusters in inland areas^16,17^. In other contexts, the United States proposes to build new urban agglomerations^18^. The experience and model of Chinese urban agglomerations’ research and construction have become a reference for global urban agglomerations elsewhere^19^. The stratified study, experience, and model of the prevalence of COVID-19 in Chinese urban agglomerations affect the thinking and direction of COVID-19 prevention measures^20,21^.

In this paper, nine representative regions of the 19 urban clusters listed in China’s 13th five-year plan (FYP)^17^ are selected. These clusters were subsequently classified into three distinctive categories of high, middle, and low levels of the category system developed by Huang and Chen^19^. To execute this research study more accurately, Pearson correlation analysis^22^ and multiple linear regression analysis^23^ in Statistical Product and Service Solution (SPSS)^24^ are used. The combined method evaluates statistical data from various variables published by the local Chinese Governments (detailed in Supplementary Appendix A). Based on the preliminary assessment of all the contextual, environmental, socioeconomic factors, the five most influential factors shaping COVID-19 prevalence across cities and regions were identified and subsequently applied in the final analysis. Details of this assessment process are explained in the later sections.

This study is mainly driven by big data analysis. We survey 45 cities in nine representative city clusters in China. The regional integration statistical data is recorded in 2019. For instance, data are extracted from the statistical bulletin published by local Chinese governments for constructing controllable variable modeling (detailed in Supplementary Appendix A). A completed multiple linear regression evaluation and best subset regression analysis are built using SPSS^25^ and R^26^, to calculate and analyze the correlation between critical variables and the COVID-19 prevalence across cities and regions. Overall, the study utilizes hierarchical modeling techniques to explore the relationship between predictable urban variables and the COVID-19 prevalence across representative Chinese cities and regions as the main objective of this study.

### Aim and objectives

This paper aims to investigate the correlation between prevalence rate of the COVID-19 pandemic and city cluster level or city level. To achieve this aim, we adopted nine representative Chinese city clusters based on the following rationales and procedures. First, due to a dearth of research, there is a pronounced need for COVID-19 infection analyses across different cities and city clusters. Subsequently, to address the research gap, we selected representative cities and city clusters and collected key relevant data for hierarchical modeling analyses. These nine city clusters were chosen based on their representativeness of city structures in China and across the world, while the city of each cluster are chosen based on the city business charm list^27^. Third, by investigating relevant factors via SPSS and Python analyses, we finalized the most influential factors in shaping the COVID-19 prevalence in the above-mentioned cities. Based on insights and evidence accumulated from the aforementioned procedures, we then examined the relationship between key factors and the prevalence of COVID-19 across cities and regions, paving the way for the final modeling analyses. To finalize our study, we completed all analytical procedures and reported results from the hierarchical modeling and city cluster analyses.

## Literature review

### Explanation of the variables studied in this paper

Based on the geographic image publishing of the collected city-level data and the associated city release information^28,29^, the infection rate of the covid-19 virus varies in the values reported by the regional slices in each city level. Therefore, studying indicators in different cities has a key impact on the relationship between the formation and pattern of response to global COVID-19 cases.

In this study, socio-economic status is one of the urban characteristic factors studied concerning the COVID-19 prevalence. According to data-driven studies by Hawkeins et al., socio-economic status plays an important role in the prevalence and mortality of COVID-19 in the United States^30^. Thus, socio-economic factors should be considered when implementing public health tools to address differences in COVID-19 prevalence in different urban communities. The literature review survey and data analysis by Mamelund et al. indicated the results of the pandemic vary depending on the income and socioeconomic status of the city's residents^31^. In a meta-regression study of the socioeconomic characteristics of the COVID-19 prevalence/mortality in the top 50 major U.S. cities, Takagi et al. argued that the COVID-19 prevalence might increase with economic factors, such as unemployment and poverty rates^32^. On the other hand, other factors cannot be neglected as well. For instance, tourism flow in different regions is associated with COVID-19 and other urban characteristic factors. A multiple regression analysis by Farzanegan et al. shows that countries facing high tourism flows are more vulnerable to cases and deaths caused by the COVID-19 pandemic^33^. Also, it was shown in Gössling et al.’s study (2020) that as the virus accompanied the flow of tourists from China to countries around the world, imported cases for COVID-19 surged, reinforcing the physical distancing and travel restrictions^34^.

Another view that has been widely discussed in many studies is home confinement or isolation, especially during the lockdown periods. This has proven to be an effective way to reduce COVID-19's spread in cities^35^ and minimize frequent movement between cities. This is based on an overarching consideration in limiting the speed at which the virus can spread. In the study of pop migration into COVID-19 epidemic modeling by Chen et al., it was found that asymptomatic infections and population movements can play a key role in the spread of disease^36^. One study by Shi and Fang looked at how human mobility from Wuhan facilitated the spread of the COVID-19 to other cities in China^37^. Their study found a decrease in the cumulative incidence of COVID-19 cases in other cities after the Wuhan travel ban. At the same time, the study shows that human mobility has a more lasting impact on provinces closer to Wuhan and with more tourists from Wuhan, but less on provinces with economic advantages^37^. Another report found a significant positive correlation between the frequency of flights, trains, and buses departing from Wuhan and the number of COVID-19 cases and cumulative numbers per day in other cities^38^. Therefore, the transportation/mobility relationship between cities and Wuhan is also a research argument focus.

According to the research of Ren et al., who had studied precautions and disinfection in hospitals during COVID-19, the transmission mode of COVID-19 was explicitly mentioned. Ren et al. (ibid) said that the most common way of transmission of COVID-19 is through airborne contact^39^. When a patient with COVID-19 enters a hospital, the COVID-19 virus may remain in the facility for a long time and remain infectious while they are alive (ibid). Since the main mode of transmission of COVID-19 is airborne, population density and movement have a significant impact on the COVID-19 pandemic. Similarly, Han et al. studied the COVID-19 developments in Beijing^40^. Their study analyzed the impact of COVID-19 in terms of population density, places of access, and number of migrants. They noted that places with high population density, such as hospitals and shopping malls, have a significantly higher incidence of COVID-19 patients. A key finding from their studies shows the impact of the number of migrants, especially those traveling from Wuhan and nearby areas, on the prevalence of the pandemic.

Based on existing scholarly work mentioned above and limitations on data availability, we verify the parameters selected in this paper are ‘city tourism development index’, ‘city economic development index’, ‘traffic distance between the city and Wuhan’, ‘urban population density coefficient’, and ‘city migration flow index during the spring festival’.

### Future research directions and considering other factors

In this paper, we mainly study the influence on the distribution and incidence of COVID-19 based on five factors: economic, urban tourism, migration flow, urban population density, and traffic distance. There are indeed many other factors which can influence the distribution and incidence of the COVID-19. For these other influencing factors, we can call them the irrelevant variables of this paper. For example, Tian et al.’s research (2020) studies the treatment and transmission of COVID-19 patients who already had some diseases^41^. Their results show that patients with SARS-COV-2 or cancer had a significantly increased probability of contracting COVID-19, suggesting that some diseases played a decisive role in the infection rate of COVID-19. Similarly, the research studies of Şahin and Jahangiri et al. highlight how the weather conditions affect the transmission of COVID-19^42,43^. The latter study has a more specific research purpose, specifically on the impact of ambient temperature on COVID-19. The results of this research showed that the transmission of COVID-19 was not strongly correlated with ambient temperature, and the experimental results can not indicate that the transmission rate of COVID-19 in areas with high temperatures is higher than that in areas with low temperature^43^. Based on the research of Şahin, the studied weather conditions are temperature, dew point, humidity, and wind speed^42^. Nonetheless, expect for wind speed, the other three kinds of weather conditions did not have strong influence on the spread of COVID-19, and the higher wind speed a region had, the lager number of COVID-19 patients would exist. These studies highlight some extraneous variables that could be considered unrelated to the pandemic prevalence. Thus, there are also a large number of these irrelevant variables in this study. Consequently, to enhance the purpose of the study, we conducted a systematic analysis according to the five selected relevant variables, so as to demonstrate the rigor of our findings.

The sudden outbreak of disease transmission can be a hostile nemesis to our cities and communities, especially if a city lacks preparedness and resilience^44^. To prevent and control the early infection of COVID-19 in the city, and to reduce the impact on the city’s economic development and humanity, and social interaction, a series of complex and multivariate prevention solutions and strategies need to be adopted^45,46^. Cities (such as Wuhan) often implement specific anti-epidemic measures as a whole to implement overall blockade or restrict the spread of COVID-19 in the flow of people^47^. However, due to the widespread impacts of the COVID-19 pandemic, it is imperative to study factors instrumental in shaping COVID-19 prevalence at the city level^48^. Insights gained on these factors can inform policymakers to develop evidence-based and effective policies to control and contain COVID-19 transmissions (and future disease outbreaks), and in turn, prevent avoidable human, social, and economic losses due to inadequate prevention measures.

As the focus of the study, we argue that ‘urban agglomerations’ cover dozens of cities and are a form of the advanced stage of urban development. Social and economic connections between different levels of cities constitute a complex network, which brings significant challenges to the work of urban agglomeration research^49^. The outbreak of COVID-19 has a potential impact on the promotion of digitalization and systems thinking in the cities and clusters systems^50^. Using big data to drive and establishing mathematical models to propose new concepts and theories for the prevention of COVID-19 in cities is a recent emerging research direction^51,52^. A closer look at the existing literature shows that most studies investigated the COVID-19 transmissions at the city level based on their analyses on individual cities or provinces^53,54^. However, few researchers focus on the elastic resilience for regional connections between different cities in regional blocks. Seminal research in the field has shown that the spaces of urban agglomerations of different scales have the hierarchical relation of boundary delimitation, mutual recognition, mutual restriction, mutual feedback, and mutual regulation^55^. “Urban agglomeration” in China refers to the geographical concept with a central city as the core and surrounded by a group of similar major cities, as well as the interaction between administrative, transportation, economic and social fields^56^. In the literature research on urban agglomerations in Western countries, an urban agglomeration is defined as the “metropolitan area”, including the peripheral areas of urban area, towns, villages^57^. Compared to other countries, the mechanism for the rise and development of Chinese urban agglomerations is much more complex and complete than that of developed countries. Overall, China’s existing (relatively mature) urban agglomeration system is an important object for studying the cross-regional propagation and hierarchical analysis of COVID-19. China’s cities have already been officially clustered into groups in the official 13th Five-Year Plan announcement of the Chinese government in 2018^58^. Thus, this study interprets and analyzes the use of big data in the identification phase of the COVID-19 outbreak in cities to find appropriate modeling methods and take control measures. By comparing and estimating the map of China’s city clusters and the COVID-infected cases, the modeling analysis of urban agglomerations is further expanded, and detailed variables are analyzed. This approach provides a clear, pre-established model map for further exploration of urban indicators in high-risk COVID-induced locations. That is to conduct the longitudinal analogy between city clusters and the horizontal analogy between equivalent cities to form a unit table to comprehensively analyze the relationship between the incidence of COVID-19 and cities in urban agglomerations. It is evident that the COVID-19 pandemic has re-exposed the vulnerability of cities to the pandemic. Therefore, there is a need to understand the basic patterns and dynamics of a pandemic and prepare cities’ response and prevention, as necessary^59^. To achieve this, the paper focuses on studying various factors of urban agglomerations, cities at different line levels under the urban agglomeration classification system, and their relationship with and influence on the induced probability of COIVD-19.

## Methodology

### Methods used in this study

Based on China’s well-established urban grading system and city clusters planning, this study uses two validation methods based on multiple linear regression (MLR) and polynomial best subset regression (PBSR). Such an approach helps promote the correlation study of the urban level, cluster groups (regional blocks) between the indicator parameters, and the prevalence of COVID-19.

Multiple linear regression (MLR) is widely used in the statistical practice of several explanatory variables and one response variable for prediction analysis^60^. Multiple linear regression is linear modeling of ordinary least-squares (OLS) regression for multiple explanatory variables to explain the linear relationship of a dependent variable. Since the start of the COVID-19 pandemic, multiple linear regression (MLR) has been widely used in various cases and mortality-related inquiries analysis. For instance, Carten et al.’s^61^ study of COVID-19 cases and mobility habit of action uses multiple linear regression (MLR) to study how mobility habits affected the spread of the COVID-19 pandemic in Italy. Similarly, Rath et al. used a combination of Linear Regression and Multiple Linear Regression models to predict trends in active cases in Orissa in India over the next few days trend^62^. In another study, the epidemiology collected and compared by Qin et al. uses a multi-linear regression approach to study the effects of gender on inflammatory responses, as well as gender biases in the COVID-19 results caused by gender-specific inflammatory responses^63^. Hence, the two combined methods are recognized as suitable and accurate methods for this research study.

The authors confirm that all methods were carried out in accordance with relevant guidelines and regulations. All data are anonymous and extracted from available public databases.

### Selection of clusters and cities samples

**Figure 1:**
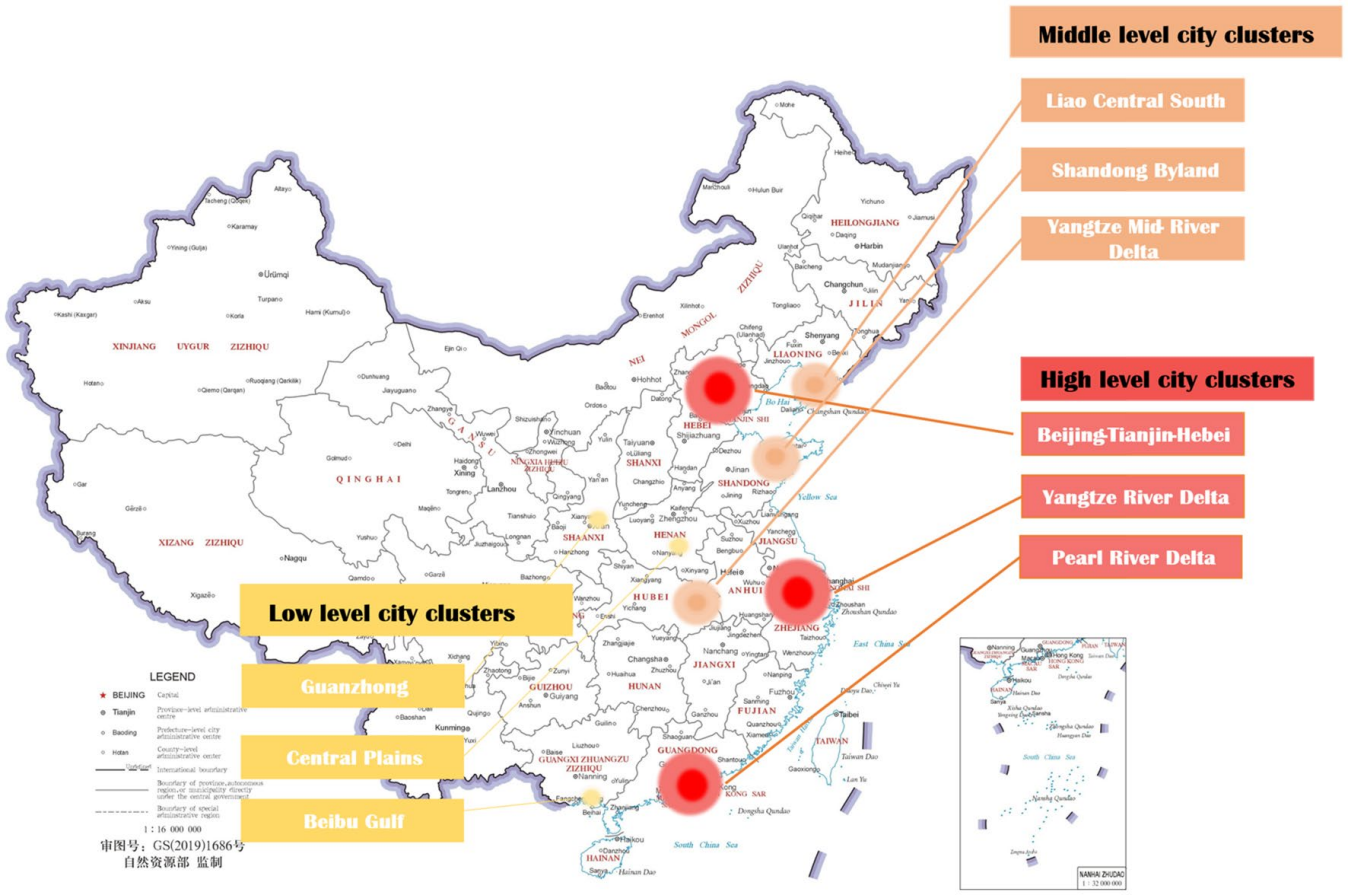
Nine representative city clusters highlighted in China’s City Cluster Plan (Source: The map of China comes from the National Platform for Common Geospatial Information Services in China. The map is generated from this available database and relevant clusters operation is completed on Powerpoint).

This paper selects nine city clusters in China (1. Beijing-Tianjin-Hebei, 2. Yangtze River Delta, 3. Pearl River Delta, 4. Liao Central South, 5. Yangtze Mid-River Delta, 6. Shandong Byland, 7. Central Plains, 8. Beibu Gulf, and 9. Guanzhong) to construct a hierarchical COVID-19 analysis and evaluation system. These selected city clusters are composed of (1) three high-level (national-sized) city clusters, (2) three middle-level (medium-sized) city clusters, and (3) three low-level (point-shaped) city clusters (see Fig. 1).

As for the definition of these urban agglomerations of different levels, the selection and classification of city agglomerations are based on the methods of ranking city agglomerations in the research by Huang and Chen^19^. The index system consisting of 10 factors and 22 indicators is constructed (Supplementary Appendix B), and Q-type clustering analysis is used to divide Chinese urban agglomerations quantitatively^19^. The results emphasize economic openness, development degree, decentralization degree, industrial structure, and resource loop effects. These five basic indicators are used for the official classification of China’s urban agglomerations. The relevant rank grading details are recorded in Supplementary Appendix A. According to their index system that utilizes five basic characteristics values to analyze 19 city clusters, nine city agglomerations with average scores of three levels, i.e., high, middle, and low, are selected. The selection method is based on the five characteristics of urban agglomerations.

The city sample selected in this research study is based on the city cluster taking a city with the highest tier or equivalent. For each city cluster, we choose five cities for data simulation calculation. The tier classification of Chinese cities in this paper is based on the online media company, First Finance^27^, developed based on collected data from 170 consumer stores and 18 head Internet companies and agencies in various fields. The investigation process evaluates 337 Chinese cities according to the five dimensions of commercial resource concentration, urban pivotability, urban vitality, lifestyle diversity, and future plasticity. Its final specific list of cities is divided into first-tier cities (or 1+), new first-tier cities (or 1), second-tier cities (or 2), third-tier cities (or 3), fourth-tier cities (or 4), and fifth-tier cities (or 5). The selection of sample cities in each cluster follows the ranking order of the provided list by the Chinese First Finance (Yicai, 220). This means we follow a consistent selection process for all nine clusters, each with the selection of the highest city level (the highest-ranked city), the highest-ranked city in the next tier (if not, the best-ranked city in the next tier), the next tier (if the first city is chosen, the second city), until we have five cities in the cluster. In this selection, we also considered data availability of the city, ensuring that the selected cases are not randomized and follow the same process. High-level clusters are consistent in tier-level selection (i.e., 1+, 1, 2, 3, 4), while middle-level clusters only have one city in mid-level (i.e., 1, 2, 3, 4, 5*). Nonetheless, the selected three mid-level city clusters are the only ones with adequate data and considered as most eligible for the study. In the low-level clusters, the pattern differs slightly depending on two factors of cluster importance and data availability (i.e., 1/2, 3, 3/4, 4, 5). However, the selection method remains the same. It is important to note that not all low-level city clusters have major cities in the upper tiers. Thus, our selection enables a consistent selection process to include a range of city tier samples in each cluster.

### Data extraction and independent variables selection in assessment

The period we study the prevalence of the COVID-19 in selected regions (city clusters and cities of multiple levels) is the half-year period from 31 December 2019 until 30 June 2020. This is based on the time the Chinese government first reported an outbreak of COVID-19 in the original epicenter, Wuhan^64^. For prevalence statistics and calculations, we surveyed all local cases for the 6 months up to 30 June 2020 and divided them by the local population to obtain the value. All data are publicly available and are extracted from the main website of the COVID-19 real time track in China^65^. All data sources are official and are suitable for data collection process. In our selection process, we have included only mainland Chinese clusters. We have excluded Tibet due to their low number of cases, and Hubei province as the original epicenter of the pandemic. Therefore, when considering the relevant factors of COVID-19, we did not choose Wuhan and Hubei Province. Correspondingly, we choose the straight-line distance of each city to the epicenter as a variable to this study.

### Urban factors source and explanation

Most of following the data sources of these seven variables are based on the 2019 National Economic and Social Bulletin in each city published by local governments in China, which is recorded in appendixes. These are the official reports published every year. For a small number of cities, certain data is missing in the official gazette, but we have found adequate corresponding data in the official China Bureau of Statistics or the local official Statistics Bureau. There are only two data sources that we cannot find at all, but we have a reasonable estimate from cities in the same province or similar areas. More detailed data and data sources are available in the provided Supplementary Appendix. To summarise, the urban factors of the study are: (1) ‘Urban population ratio’, to measure the process of urbanization; (2) ‘retail sales of consumer goods’, to measure the circulation of goods shopping in the city, which may increase courier infections; (3) ‘total number of tourists and (4) ‘tourism Income’, both are used to measure the tourism attraction of one city to see if tourism industries bring negative effect in this pandemic; (5) ‘jobless rate (%)’, to measure whether jobless and/or homeless people increase the likelihood of a pandemic spreading; (6) ‘proportion of people sharing one health facility’, to evaluate the quality of local health facilities, which is measured by dividing the number of health facilities by the local population; and (7) ‘urban per capita GDP’, which it is obtained by dividing the total population by the GDP of the city in 2019 and used to measure the degree of economic development maturity of the city.

The urban population density in this study refers to the density of people living within a city. By using the record data of the Statistical Yearbook of Chinese cities 2019, the calculation formula for the urban population density factor is recognized as the ratio of city population over the overall city area. The index data for the mobility scale are derived from Baidu Migration’s public data. In order to explore the prevalence data more accurately, we only collect the scale data for the statistical movement/mobility at the city scale. The migration flow index data are obtained from Baidu Migration Database for the period between January 10 to January 23, 2020, which is considered a crucial time before the Chinese New Year Holidays (or commonly known as the Spring Festival) and the lockdown of the original epicenter, the City of Wuhan (i.e., on 23rd January 2020). The sum value of that period of the scale coefficient of mobility is used as the index of the flow of people moving into each city during the spring festival season^66^. The proportion of the elderly population over 60-year-old in the city is the result of China’s main data of the Seventh National Population Census. The straight-line distance from the original epicenter Wuhan to the cities is extracted from the distance database between cities in China^67^.

**Figure 2:**
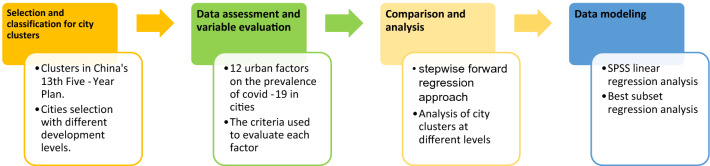
Overview of methodology and model analysis.

Table 1 Summarizes the selected city clusters and cities in each cluster. Figure 2 summarises the methodological framework of the study.

**Table 1:**
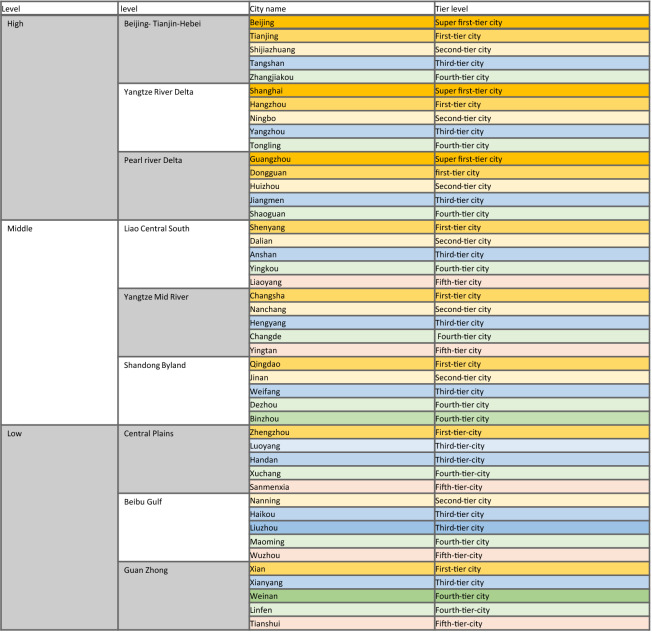
The urbanization hierarchical structure of nine selected city clusters in China, three per level (high, medium, and low), including five cities per city cluster.

### Main method and analysis

#### Basic multiple regression analysis method

**Figure 3:**
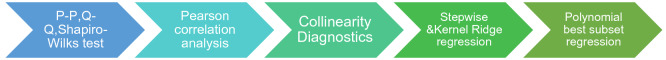
Flow chart of multiple linear regression analysis.

To compare the basic original data, two methods of scatter diagram and normal distribution tests are first used to ensure the correctness of data assumptions. Therefore, after taking logarithms of some data, the Shapiro–Wilks test is adopted to ensure that data are normally distributed, and there is no multi-collinearity. Then we perform a basic Pearson correlation coefficient test. When obtaining the basic Pearson correlation coefficient, we find a large R squared value of correlation between some independent and independent variables. Collinearity diagnostics is used to test data with multiple collinearities between them. In order to eliminate the influence brought by some variables with strong collinearity, stepwise forward regression approach and Kernel Ridge regression are successively used to improve the data deviation, so as to make the final model more consistent with the actual regression equation. In addition to these two basic common statistical methods, we also use more accurate full subset regression and the best subset method for screening. The flow chart process is summarised in Fig. 3, highlighting all five steps for this proposed multiple regression analysis methods.

#### Stepwise forward regression and Kernel ridge regression

For the analysis of the prevalence of COVID-19, when using the method of stepwise forward regression, we use the Shapiro–Wilks test method, P–P, Q–Q graph to test the normal distribution of the data. Due to the multiple collinearities of the arguments, stepwise forward regression eventually only selects three indicators to model urban prevalence: PGDP, DRW, and LMS. They establish a four-dimensional variable relationship with LPV. These filtered variables’ inflation factor (VIF) values are all less than 2, and they explained 57% of the model variation.

As all city indicators have certain correlation, some inconsistent variables may be excluded from the above process. Kernel ridge regression is essentially an improved least square method, which is specially used for collinear data analysis of biased estimation regression method. But at the same time, its disadvantage is that it gives up the unbiasedness of the method of least squares and loses part of the information. This occurs at the cost of giving up part of the accuracy, but it can seek the regression equation with less effectiveness and more consistantency with the reality. Ridge regression can be applied between LMS and LTN, LRS, PGDP. For instance, the migration scale of population and the correlation between tourist flow are highly correlated. In this way, some variables are not discarded when included in the study. In addition, these key variables were identified and validated through principal component analysis, hierarchical analysis steps, and parametric regression models. These are established analytical methods to study the prevalence of COVID-19.

#### Polynomial best subset regression (PBSR) analysis

**Figure 4:**
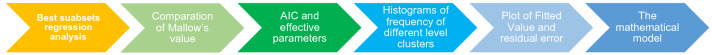
Flow chart for best subset regression analysis by R.

Full Subset regression can fit all possible combination models of predicted variables, and then screen out the best model under existing variables according to R^2^, corrected R^2^, Mallow’s value, Cp, and AIC. This process is called ‘best subset selection’. PBSR analysis can fit and test all possible combinations of the parameters and select the best subset solution^68^. As the interactions among multiple parameters may have additional implications for the prevalence of COVID-19, PBSR analysis can investigate the overall correlations and makes up the limitations of Stepwise Forward Regression and Kernel Ridge Regression approaches, which can find a balance between modeling in various ways affected by errors in their respective methods (see Fig. 4).

**Figure 5:**
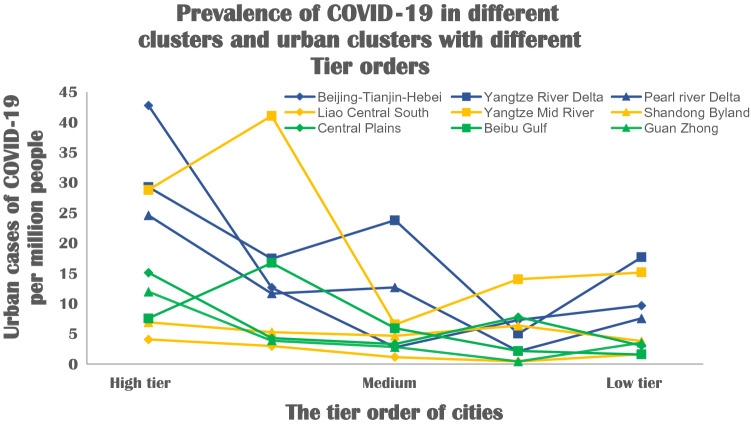
Prevalence rates in 45 cities of nine clusters and different tier level orders.

PBSR analysis is achieved in four steps, as also summarized in the flow chart of Fig. 5:

- The best mathematical model can be preliminarily chosen by comparing the value of Mallow’s ${C}_{p}$ and the value of parameter number ($p$)^69^.
- Compare the Akaike information criterion (AIC) and define the ultimate parameters that strongly correlate with the prevalence of COVID-19^70,71^.
- Modify the mathematical model by Mosteller and Tukey’s bulging rule^72,73^.
- Plot of fitted value and residual error.

Ultimately, the final mathematical model is available, and the t-testing can screen the most influential factors.

## Results and discussion

### General comparison and prevalence results

**Figure 6:**
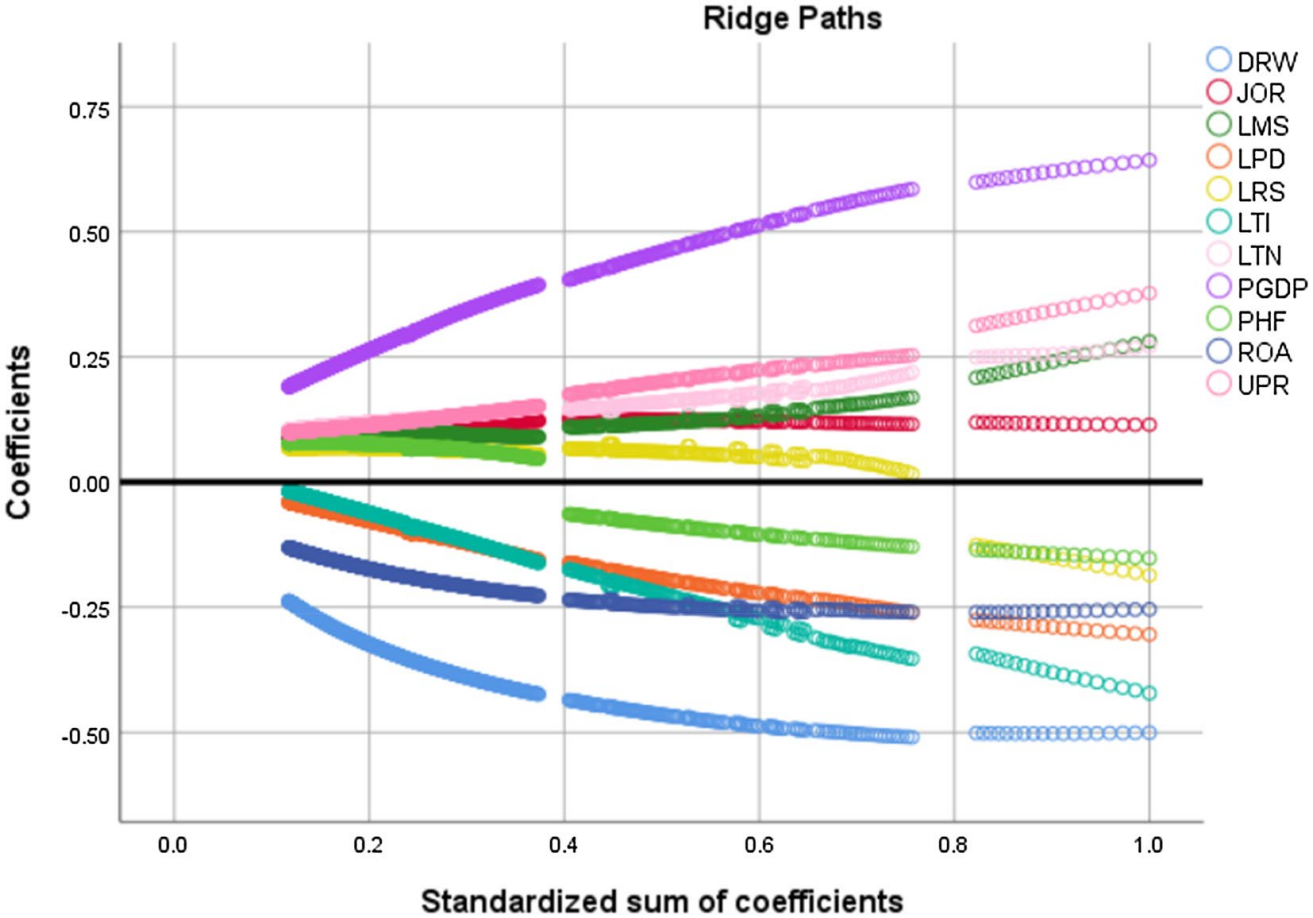
The Ridge paths of parameters.

Figure 6 shows COVID-19 infection rates of high-tier and low-tier cities in different clusters. The comparison highlights basic changes in the observed and simulated prevalence rates with changes in the city’s tier level. When comparing COVID-19 infection rates in cities at five different tiers over the period from December 29th, 2019, up to June 30th, 2020, the infected rates showed a similar trend. With only some exceptions (i.e., Nanchang city, Haikou city), this trend indicates that cities with lower tiers had significantly lower levels of COVID-19 infection rates compared with higher-tier cities from the highest-tier level to the fourth-tier level. However, the prevalence of COVID-19 in the lowest tier cities showed a different degree of recovery in the statistical data in all three high-level clusters. The diagram of case number may suggest a correlation between the level of urban development and the level of COVID-19 pandemic infected cases.

As shown in Table 2, among the 45 sample cities, the highest average COVID-19 infected case in all highest-tier cities is 19.04. In contrast, the lowest average COVID-19 infected case in lowest-tier cities is 7.10. The average number of cases, the highest level of the city, was 171% higher than the lowest level. Comparison results of different tier levels of cities are discussed above. We note the uneven development of urban agglomerations also produces differences in the prevalence of COVID-19. This is explored further in the following analysis.

**Table 2:** Comparison of cases among different cities and city clusters.

Prevalence	High level clusters	Middle level clusters	Low level clusters	Average
Tier 1st	32.25	13.28	11.58	19.04
Tier 2nd	13.95	16.46	8.32	12.91
Tier 3rd	13.10	4.15	4.04	7.10
Tier 4th	4.84	6.96	3.47	5.09
Tier 5th	11.65	6.88	2.76	7.10
Average	15.16	9.55	6.04	N/A

In the observation and modeling plotting of the input data of 45 cities, we note that the infected cases of the high-level clusters are higher (Average number = 15.16). It is only in the 4th tier level that an abnormal number of cases appeared in the data (Tier 4th = 4.84). In contrast, mid-level clusters’ infected cases vary widely and are volatile (from 16.46 to 4.15, not relying on the tier level order, Average rate = 9.55). The results show the infected cases of COVID-19 in the low-level clusters were the lowest (Average rate = 6.04). In addition, in this low urban cluster layer, the number of infected cases decreases proportionately with the decrease of the city tier level. According to the basic representation, we hypothesized that the number of cases was related to the city and cluster’s grading system. Therefore, data collection and research were conducted on 12 independent variables, including people’s livelihood and economy, social mobility, population structure, geographical location, etc. Detailed analysis of selected influencing factors within the city cluster and predicted modeling accuracy is thoroughly analyzed in the following section.

### Multiple linear regression analysis

#### P–P, Q–Q, Shapiro–Wilks test

According to the requirements of Pearson and multiple linear regression in statistics, all variables must meet the criteria of normal distribution. Therefore, P–P, Q–Q, and Shapiro-Wilks tests are applied in data review. Due to the limited number of urban clusters in China, covering all cases with only a few samples is difficult. As a result, the overall distribution is normal, but the sample does not meet the normal distribution. Nonetheless, all parameters are processed to meet the requirements of normal distribution after log algorithm transformation, square root transformation, and reciprocal transformation of unqualified data. In the final test, the P values of Shapiro–Wilks test of all variables are greater than 0.05, which belong to normal distribution. The R studio running results are recorded and provided in the Supplementary Appendix E.

#### Pearson correlation

Pearson correlation is used to evaluate the degree of correlation between selected influencing factors in cluster cities and the prevalence of COVID-19 (Sweet, 1999). Five related indicator variables were used to evaluate the development level and tier level of the city. This helps to evaluate their degree of correlation of COVID-19 prevalence. The correlations between various coefficients were identified using the Pearson correlation analyses as the flowing Table 3 shows.

**Table 3:**
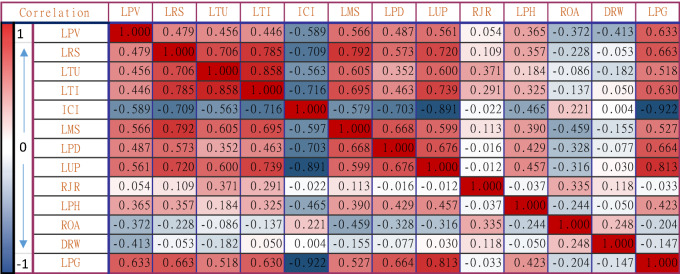
The Pearson correlation analyses between the economy, social mobility, population structure, and geographical location variables and the city level prevalence of COVID-19.

Dark color** Correlation is significant at the 0.01 level.

Moderate color* Correlation is significant at the 0.05 level.

Light color indicates ‘No strong correlation’.

According to the Pearson analysis, the prevalence rate of all cities is mainly related to several variables, i.e., LPG, ICI, LUP, and ICI. However, we also found that as these variables of economy, social mobility, population structure, and geographical location are statistically relevant urban measurement factors, they are highly correlated and may constitute multiple collinear overlaps.

#### Multiple linear regression of stepwise method

Stepwise regression methods have a reasonable independent variable screening mechanism, which can help avoid the influence of non-statistically significant independent variables on the regression equation^74^. It checks the effect on the model by adding one parameter at a time, then leaving the filtered variables that fit the dependent variables and removing the other irrelevant variables.

In the final model result, only variables ‘PGDP’, ‘DRW’, ‘LMS’ are remained in the Stepwise method model. These three variables and LPV construct four-dimensional functions in this model relationship. The stepwise method regression results show the independent variables, ‘LPG’, ‘DRW’, ‘LMS’, and the prevalence of COVID-19 ‘LPV’ are relatively close. The R2 of the final comprehensive model is 0.533 with these three indicators. Also, P < 0.01 shows that measuring the city’s prevalence can explain this prevalence at 53.3%. In the statistical data of 45 cities, after removing some variables with high collinearity and irrelevant variables, the most important correlation variable is per capita GDP income, while the mobility rate of population and the straight-line distance from Wuhan are auxiliary modification variables.

The partial regression coefficient of each variable included in the model is not zero, and the P  ≤ 0.01 for each independent variable. The final regression model in stepwise method is:

$$LPV=0.909LPG-0.000450DRW+0.468LMS-0.200,$$

$$Log10\left(Prevelance \,value\right)=0.909Log10\left(Per \,capital \,GDP\right)-0.000450\left(Distance\, from\, Wuhan\right)+0.468Log10\left(Mobility\, scale\right)-0.200.$$

The standardized regression coefficient Beta (0.909, − 0.000450, 0.468) is used to compare the influence of the corresponding variable (LPV) of different independent variables (i.e., LPG, DRW, and LMS). Under the statistical significance premise, the larger the standardized regression coefficient Beta shows the corresponding independent variable has a greater prevalence effect. In this case, under the premise of meeting statistical significance, the order of the magnitude of the effect on prevalence is urban per capita GDP, the straight-line distance from original epicenter Wuhan to this city, and Mobility scale (i.e., inbound direction) index sum from 10th Jan to 23rd Jan 2020. The lower value of VIF (< 2) indicates that the final 3 independent variables selected for modeling do not have multi-collinearity problems in observations and have substantial explanatory power to explain maximum model variation.

### Kernel ridge regression analysis

Some relevant and useful variables may be removed incorrectly due to collinearity, which cannot be effectively used to explain the LPV model. To prevent this phenomenon, we added the Kernel ridge regression analysis. Ridge regression is a regression method specially used for collinear data analysis with biased estimation. It is an improved least square method. Ridge regression reduces the coefficient by introducing a penalty term equal to the sum of the squares of the coefficients multiplied by the penalty coefficient. The coefficients range from 0 (without penalty) to 1 to correct the coefficients. According to the Ridge paths, when k = 0.25, the coefficient of the image tends to stabilize. By selecting the table data results of ridge regression analysis, we found that ‘LPG’, ‘DRW’, and ‘ROA’ contributed significantly to the model formula. When K = 0.25, the ridge beta values of ‘PGDP’, ‘DRW’, and ‘ROA’ are 0.34, − 0.39, and − 0.21. Ridge regression can only screen out these variables, which are strongly correlated with LPV. When substituted into the model fitting, the P-value is less than 0.05, indicating that these variables can be explained reasonably.

### Best subsets regression analysis

This paper makes the best subset regression for the separation of three different levels of urban clusters. This facilitates understanding of the true impact of the complex degree of development in different urban clusters on the prevalence of COVID-19. The regression model selection criteria and comparison method are two key steps carried out through the RStudio software. Regression using best subsets can be used to compare different regression models that include a specified subset of predictor variables.

In the hierarchical study of the city clusters, due to the separation of data, k–S, Q–Q, P–P, Shapiro–Wilk, are carried out on the urban distribution of three different levels. In the analyses, the data that do not obey the normal distribution is standardized, and finally, all data meet the requirements of the hypothesis. When testing the normal distribution of datasets of different level city clusters, it is found that all cities meet the requirements of normal distribution in the high-level city clusters. In the medium-level city clusters, in order to meet the requirement of normal distribution, we transform ‘PGDP’ by log algorithm, so that the data standardization can meet the requirement of the hypothesis. In the low-level city clusters, the log algorithm is enforced to the variable ‘PHF’ and ‘DRW’, to meet the normal distribution standard.

Subsequently, we start with a list of all possible models and constantly choose the best subset.

The value of Mallow’s ${{\varvec{C}}}_{{\varvec{p}}}$ should be greater and close to the parameter numbers, so the best model can be selected, as shown in Table 4.

**Table 4:** Mallow’s ${C}_{p}$ and parameter selections of 11 variables.

Cluster	${\varvec{p}}$	Selected p	Mallow’s ${{\varvec{C}}}_{{\varvec{p}}}$
High-level	5	‘LTN’, ‘JOR’, ‘PHF’, ‘PGDP’ ‘DRW’. LMS	0.488
Middle-level	4	‘LTI’, ‘JOR’, ‘PHF’ ‘DRW’	− 0.1436
Low-level	6	‘LTN’, ‘LTI’, ‘LPD’ ‘JOR’, ‘PGDP’, ‘LDW’	4.3701

The parameter numbers ($p$) indicates the maximal parameter number in the mathematical model.

#### Akaike’s information criterion (AIC)

At this stage, the model compares the Akaike information criterion (AIC) and resort to stepwise regression (Table 5). It defines the ultimate parameters that have a strong correlation with the prevalence of COVID-19 (Zhao, 2020).

**Table 5:** AIC and effective parameters.

Cluster	$\mathrm{AIC}$	Parameters (except prevalence)
High-level	− 34.99	LTN, JOR, PGDP
Middle-level	− 44.82	LRS, LTN, LMS, JOR, PHF, DRW
Low-level	− 52.61	LRS, LTN, LTI, LMS, UPR, JOR, PHF, ROA, PGDP, DRW

According to the AIC choosing law, the following formulas were given as the hierarchical analysis result of city clusters.

**High-level clusters:**

$$\left(LPV\right)=2.0797-0.5525\left(\mathrm{LTN}\right)+0.1410\left(\mathrm{JOR}\right)+0.0063\left(\mathrm{PGDP}\right).$$

Adjusted R-squared: 0.4233.

**Middle-level clusters:**

$$\left(LPV\right)=2.1072-0.5940\times \left(LRS\right)+0.6258\times \left(\mathrm{LTN}\right)+0.6185\left(\mathrm{LMS}\right)-0.2871\left(\mathrm{JOR}\right)+0.0002\left(\mathrm{PHF}\right)-0.0007\left(\mathrm{DRW}\right).$$

Adjusted R-squared: 0.86.

**Low-level clusters:**

$$\left(LPV\right)=-5.4356+1.021\left(\mathrm{LTN}\right)-1.1859\left(\mathrm{LTI}\right)+0.6964\left(\mathrm{LPD}\right)+0.1385\left(\mathrm{JOR}\right)+0.0130\left(\mathrm{PGDP}\right)+1.541\left(\mathrm{LDW}\right).$$

Adjusted R-squared: 0.8405.

T-testing indicates that the mathematical model would be correct when the value of ${{\varvec{P}}}_{{\varvec{r}}}$ is larger than the value of $\left|{\varvec{t}}\right|$. Therefore, the smaller the value of ${{\varvec{P}}}_{{\varvec{r}}}>\left|{\varvec{t}}\right|$, means the greater influence of the parameter on the prevalence of COVID-19. Furthermore, according to the t-testing result, the most influential parameter in the high-level cluster is PGDP, and its value of ${{\varvec{P}}}_{{\varvec{r}}}>\left|{\varvec{t}}\right|$ is only 0.118. The relative influential factor in the middle-level cluster is DRW. At this cluster level, all three clusters almost have the closed geography place weight in the correlation with the prevalence of COVID-19. In the low-level cluster, the influence factors are PGDP, LDW, LPD, LTI, and LPD.

## Discussions and limitations

After analyzing the infected rate of COVID-19 at multiple scales and levels, we found differences in results between cities and clusters. To understand several important triggers for COVID-19 infection cases in the structure of different urban clusters, the corresponding regional cluster case analyses apply multiple linear regression analysis. China’s 19 city clusters have hierarchically distinct structures. There are differences in the number of COVID-19 cases between different urban clusters and the city levels, indicating that variations at the regional level were found to be distributed. The primary differences included different economic factors, social mobility, demographic factors, and geographical factors. Although differences in the same city clusters are not significant, urban-level differences are most obvious in different levels of city clusters. Therefore, we should not only focus on the study of the infection spread and the prevalence of the disease in a single city or a single province. Hence, it is vital to consider such analyses at the city cluster level, representing regional urban hubs.

The results verify that the prevalence of COVID-19 in different urban clusters. It is significantly higher in high-level clusters and lower in low-level clusters. Among them, the prevalence rate of urban clusters in Yangtze Mid-River is generally higher than that of urban clusters at the same level, which is because of the fact that Wuhan is in the center of the urban cluster, thus causing the high prevalence of the whole region. Statistics show that the population-based prevalence of COVID-19 has a better effect on a place than the number of cases alone. This fact is also verified in the process of assessing the correlation between these variables and the prevalence of the disease. The combined Stepwise method and Kernel Ridge Regression analysis results show that of the 12 variables identified, urban per capita GDP (R^2^ = 0.646), the straight-line distance from the Wuhan to this city (R^2^ =  − 0.413), the mobility scale during the spring festival (R^2^ = 0.566), the ratio of the elderly population in this city (> 60-year-old), (R^2^ =  − 0.372) are the most relevant variables in evaluating the prevalence in regional cities.

In the four significant variables analyzed, almost all city clusters’ urban per capita GDP was significantly associated with COVID-19 infections. For instance, in the low-level city clusters, this correlation was found to be quite high. This finding shows that the different degree of urban economic development partly explains the inducing factors of COVID-19. It is also identified that the most influential factor in the prevalence of high-level city clusters is the higher-level mobility scale during the spring festival. This finding highlights the significance of the regional economic level and demographic structure of the city of high-level clusters in the process of fighting COVID-19 prevalence. Hence, the degree of differentiation is not high, and the effect of the mobility scale (i.e., inbound mobility) during the spring festival on the prevalence of COVID-19 was significant. The ratio of the elderly population in this city (> 60 years old) has a weak correlation with the prevalence of COVID-19 at the city level. Still, it also plays an important role in the analysis of ridge regression. The straight-line from the original epicenter Wuhan to this city is proven to be a significant geographical factor, indicating the impact of the geographical location of the city cluster near Wuhan is even more significant.

The best subset method finds a correlation between different influential variables and three levels of clustering. To clarify the cluster-level details, hierarchical analysis helps to explore the final research formulas for urban agglomerations at different levels. The results show that the prevalence of high-level clusters is closely related to per capita GDP, but the adjusted R^2^ of the final model is 42.33%. The remaining relevant variables are ‘jobless rate’ and ‘tourism income’. Both variables are related to the economic and social assets of the city. This analysis suggests that economic prosperity may have created a higher degree of COVID-19 transmission in higher-level regions and city clusters. In the model analysis, the city’s economic development index and the per capita GDP are the most influential factors among all high-level city clusters. Jingjinji, Yangtze River Delta, and Pearl River Delta have the highest COVID-19 transmission rates and are home to China’s most significant economic volume. Interestingly, these clusters overlap with China’s clusters of most developed cities at the national level. These combined insights suggest that economic factors are crucial indicators and can serve as possible proxies in gauging various cities’ vulnerability to the COVID-19 transmission and faster rate of COVID-19 prevalence at the regional level.

The prevalence of medium-level city clusters is closely related to the straight-line distance from Wuhan. Among them, the results indicate that the prevalence of middle-level city clusters is no longer determined by the degree of local economic development. The results also need the geographical distance from Wuhan as supplementary factors. Because most of the mid-level city clusters are geographically close to Wuhan, the scale of population mobility during the Spring Festival transport in the city, and the straight-line distance between the city and Wuhan appeared more significant (P < 0.001). The adjusted R^2^ of the final model is 86%. In contrast, the city-level tourism income, the city-level population density, and the other six factors are not closely related to this evaluated model.

The prevalence of cases in low-level clusters is closely related to the per capita GDP, the straight-line distance from Wuhan, and the population density. This finding is because the cluster of small cities is fragile. Hence, there are more inducing factors. For instance, the smaller cities had to respond to the COVID-19 pandemic with more stringent quarantine measures during the outbreak waves. The differences of economic factors in small urban agglomerations decrease, so it is difficult to intuitively measure the prevalence rate of COVID-19 in low-level city clusters by the economic income alone. In some underdeveloped cities and regions, we verify they are not in close contact with the external economy due to their underdeveloped economy. Therefore, the difference in the prevalence of COVID-19 between them is not intuitive in terms of economic factors. In relatively underdeveloped areas, population migration and the size of the region's population density have an intuitive impact on COVID-19. In mid and low-level urban agglomerations or city clusters, their variable factors seem to be more consistent with the impact of the prevalence of COVID-19, with higher regulation of the adjusted R^2^ of 86% and 84%, respectively.

### Limitations

The data analysis and fitting model method is suitable for adopting more impact factor analysis for COVID-19 prevalence and applying it to the similar cluster-cities structure in other countries. The study’s findings provide sample calculation methods for predicted COVID-19 modeling in city clusters to help policymakers take precautions before the COVID-19 outbreak. While Chinese city clusters are just used as examples, the findings of the current study can inform research in international contexts as well. The hierarchical model discussed in the current study was developed through multi-procedure modeling analyses and validated rigorously by mathematical models. The differences between developed and underdeveloped areas, for instance, are verified through city analysis in each cluster. Through inter-cluster and intra-cluster analyses, the study helps generate a data-driven analysis of the prevalence of COVID-19 in representative Chinese cities and regions.

However, there are some methods limitations to this study. For example, in selecting possible factors, the literature is based primarily on government reports, which are more relevant to actual factors. All these data are extracted from official data. In the study of the variables in the model, we found that there is a significant Pearson correlation between some variables. Although these factors are not common, there is still a strong correlation between them. Therefore, we made a ridge regression to make up for it. In addition, the stepwise method and best subset method are given a simple regression formula, which is the general formula for all urban clusters and a single formula for the hierarchical cluster level. Therefore, the results may be general, which is in line with the purpose of this study, i.e., to study the effects that can be achieved by demonstration calculations. We note that it is difficult to obtain accurate individual analyses for each city cluster. Thus, more data would be preferable to study the calculations more accurately. Although our goal is to obtain the most reliable available data for advanced prediction, this study is limited based on the number of city clusters and the limitations of the data disclosure. Regarding data, we note the use of 2019 data for tourism income, which is modeled to be each city’s scenario of tourism flow effect. Therefore, it is inevitable that tourism revenue figures for the first half of 2020 are lower than the same period last year because of the pandemic. For instance, according to iiMedia Research (2020), the COVID-19 pandemic led to an immediate suppression of aggregate social demand, such as tourism, catering, and transportation^75^. Similarly, we used 2019 data for population density and per capita GDP. This is verified by visible trends available in the past few years. Lastly, we confirm that the selected parameters are required to be uncorrelated. If the parameters are correlated to each other, they may have implications for results. In a real case scenario, multiple related parameters can affect the results (Cheshmehzangi, 2020). For instance, in the process of correlating the function, the method is not highly sensitive to the power of the parameters (i.e., function images written by x^2^ and x^4^ have little difference under the existing data), which could lead to errors in predicting the results. However, the paper’s data analysis tried to minimize such errors by conducting multiple models through relevant modeling tools.

## Conclusions

This study examines factors that impact COVID-19 prevalence across different Chinese cities and city clusters. By using rigorous multiple regression models and related best subset selected methods, we verify how the structures of different urban clusters regulate their indicators in relation to the prevalence of the COVID-19 pandemic. In this paper, after considering various factors, we found divergence in inducing factors of prevalence in different city clusters. The difference can be summed up as ‘economic factors’, ‘social consumption factors’, ‘population mobility scale factors’, ‘population age structure factors’, ‘urban structure factors’, and ‘geographical factors’.

Of all the 45 selected cities of nine urban agglomerations, GDP per capita (R = 0.646) has the highest correlation with the prevalence of COVID-19. This is attributed to the fact that areas with high economic development are related to various comprehensive indicators such as ‘population flow’ and ‘density’, with the highest impact on the final results. In addition to applying the findings of our study to cities with known infections, researchers can also utilize this paper's insights to evaluate not-yet-infected cities’ vulnerability to COVID-19. As indicated above, investigation of cities’ economic factors alone has the potential to help researchers quickly determine which ones are most vulnerable to pandemic spread. From the results, the other two main variables are the geographical location of different cities to Wuhan City (R = 0.0.413) and the scale of population mobility/movement during the Spring Festival (R = 0.566). However, local governments can be guided by the need for restrictions on population mobility and travel bans to effectively limit the COVID-19 pandemic in economically prosperous areas, particularly in regions or cities closer to the new origin of the outbreaks. This has been the case for the later smaller outbreaks of the COVID-19 at the city or regional levels. After counting the effects of these three variables on 45 cities throughout the region, the effective description rate of fitted R^2^ reached 53.3%.

To summarise, in high-level city clusters, the degree of regional economic development appears to measure their vulnerability to COVID-19. In middle-level city clusters, the vulnerability to COVID-19 cannot be assessed by the economic development level of the region alone, but by the geographical location of the city cluster and the origin of the epidemic, and the scale of population mobility rate. In low-level city clusters, the economic development level of the region is relatively slow, and all other factors are relatively backward and/or lower. Therefore, it is not possible to provide a perfect assessment of their susceptibility to COVID-19 from the factors of one or two cities alone. As well as the scale of population movement rate, regional tourism development, health measures, and other factors are also worth considering in future research studies.

These combined insights suggest that, by mitigating potential differences in research contexts, this model, along with our study’s findings, can be justifiably applied across different city clusters. Overall, the study can help health experts and government officials formulate evidence-based and effective public health strategies that can control the spread of COVID-19 and prevent future pandemics.

## Supplementary Information

Supplementary Information 1.

Supplementary Information 2.

## Abbreviations

LPV: Prevalence value after log algorithm transformation
LRS: Retail sales of consumer goods after log algorithm transformation
LTU: Total number of tourists after log algorithm transformation
LTI: Tourism income after log algorithm transformation
ICI: The reciprocal of real disposable per capita income
LMS: Mobility scale (direction: in) index sum after log algorithm transformation
LPD: Population density after log algorithm transformation
LUP: Urban population ratio after log algorithm transformation
RJR: The square root of jobless rate
LPH: Proportion of people sharing one health facility after log algorithm transformation
ROA: Ratio of the elderly population (> 60 year) in this city
DRW: The straight-line distance from original epicenter Wuhan to this city
LPG: Urban per capita GDP after log algorithm transformation
