# Supplementary material for: A hierarchical study for urban statistical indicators on the prevalence of COVID-19 in Chinese city clusters based on multiple linear regression (MLR) and polynomial best subset regression (PBSR) analysis
Source: Sci Rep. 2022 Feb 4;12:1964. doi: 10.1038/s41598-022-05859-8 (PMC8817036; doi:10.1038/s41598-022-05859-8)
Supplement: Supplementary file 2 — Supplementary Information 2. [file 41598_2022_5859_MOESM2_ESM.docx]

**Appendix A**

Table S1. Original Government links from Chinese Websites – used for all selected case studies


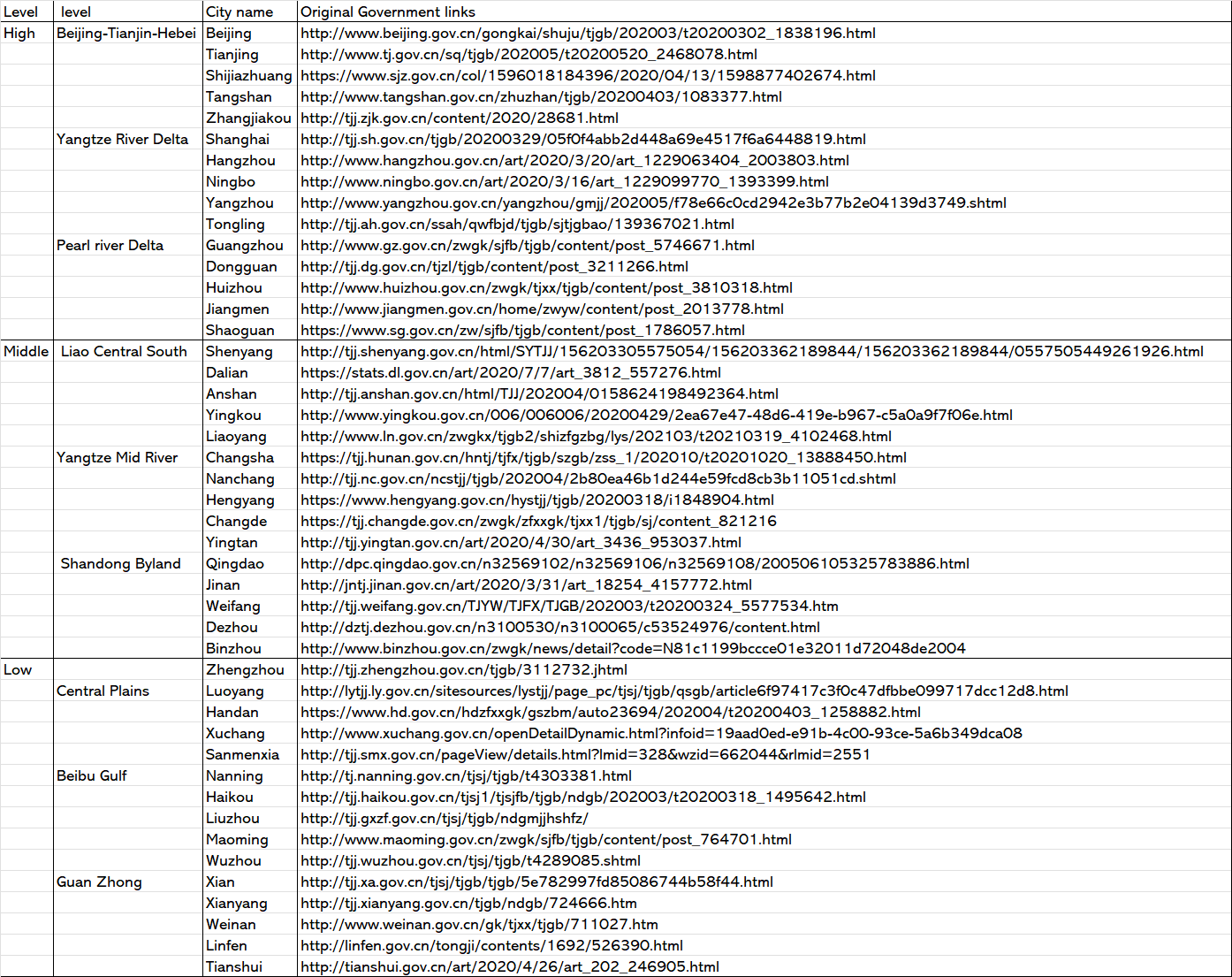


**Appendix B**

Table S2 The index system consisting of 10 factors and 22 indicators

| Level | City  cluster name | Economic  openness | Development  degree | Decentralization degree | Industrial structure | Resource loop effect |
| --- | --- | --- | --- | --- | --- | --- |
|  | **Index property** | **Positive** | **Positive** | **Negative** | **Positive** | **Negative** |
| High | Yangtze River Delta | 80.63 | 100 | 24.95 | 80.74 | 75.98 |
|  | Pearl river Delta | 100.00 | 98.90 | 4.59 | 100.00 | 73.13 |
|  | Beijing-Tianjin-Hebei | 51.81 | 56.25 | 17.03 | 90.49 | 67.99 |
| Middle | Yangtze Mid-River | 21.99 | 40.20 | 47.41 | 38.43 | 30.28 |
|  | Shandong Byland | 30.37 | 48.31 | 0.00 | 57.53 | 62.85 |
|  | Liao Central South | 49.38 | 37.57 | 10.85 | 34.15 | 59.07 |
| Low | Beibu Gulf | 11.50 | 15.49 | 59.24 | 43.57 | 34.60 |
|  | Qianzhong | 2.55 | 0.00 | 11.18 | 22.76 | 50.18 |
|  | Guangzhong | 11.19 | 22.03 | 29.24 | 52.04 | 47.39 |

**Appendix C**

Table S3. The recorded and data source data source


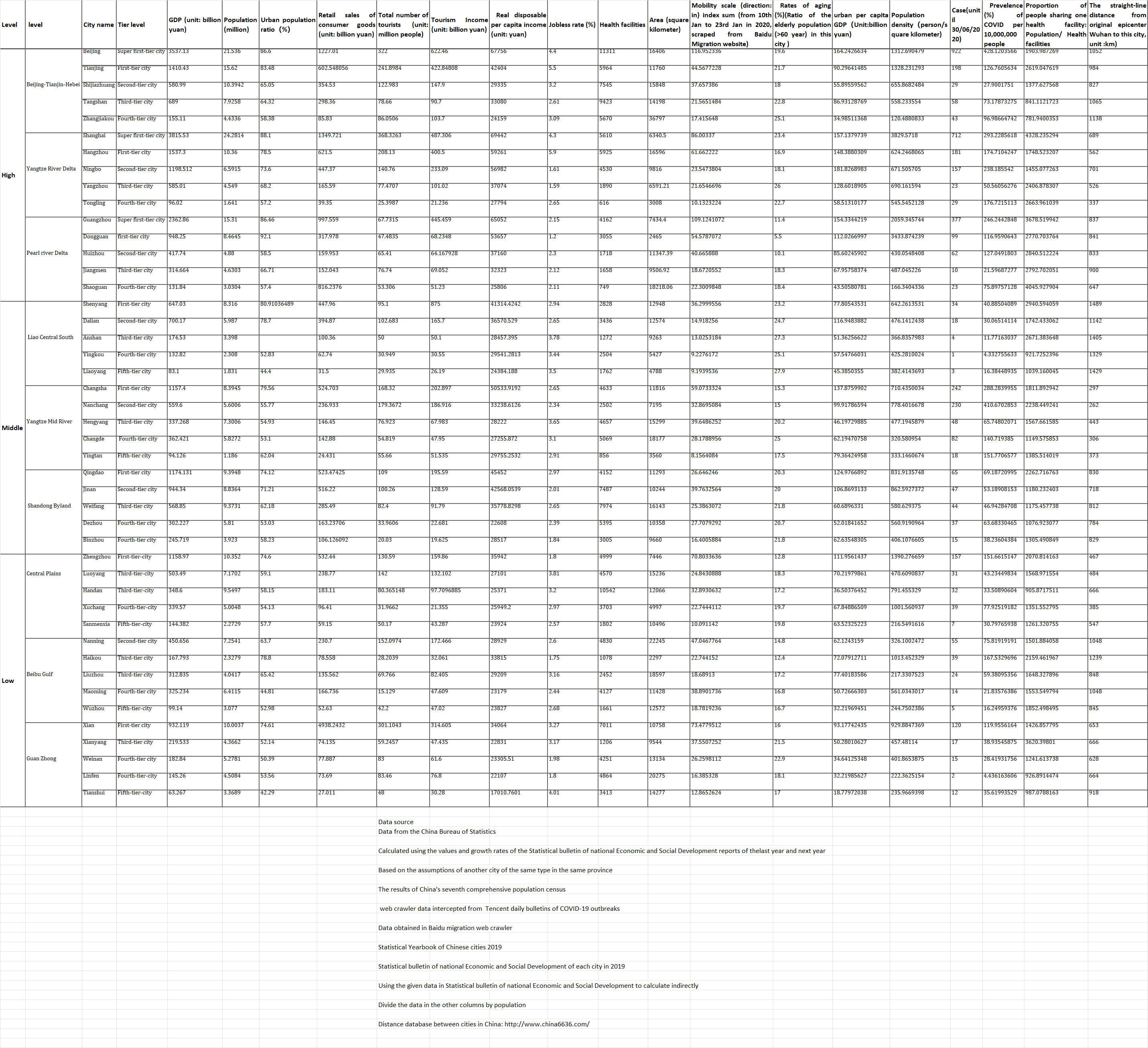


**Appendix D**

**Mallows** $\boldsymbol{C}_{\boldsymbol{p}}$

Table S4. Mallow’s $C_{p}$ and parameter numbers in 3 levels cluster

| High level cluster | | | | | | | | |
| --- | --- | --- | --- | --- | --- | --- | --- | --- |
| $\boldsymbol{p}$ | 1 | 2 | 3 | 4 | 5 | 6 | 7 | 8 |
| Mallow’s $\boldsymbol{C}_{\boldsymbol{p}}$ | -6.027 | -4.461 | -3.058 | -1.244 | 0.488 | 2.293 | 4.171 | 6.108 |
| Middle-level cluster | | | | | | | | |
| $\boldsymbol{p}$ | 1 | 2 | 3 | 4 | 5 | 6 | 7 | 8 |
| Mallow’s $\boldsymbol{C}_{\boldsymbol{p}}$ | 1.049 | -2.591 | -1.594 | -0.1436 | 1.180 | 2.699 | 4.369 | 6.134 |
| Low-level cluster | | | | | | | | |
| $\boldsymbol{p}$ | 1 | 2 | 3 | 4 | 5 | 6 | 7 | 8 |
| Mallow’s $\boldsymbol{C}_{\boldsymbol{p}}$ | 15.0827 | 12.4295 | 9.8450 | 5.7611 | 5.4281 | 4.3701 | 5.8489 | 7.3891 |

**P, T values**

Table S5. The results and relative coefficients of each clusters are summarized

| High level cluster | | | | |
| --- | --- | --- | --- | --- |
| Parameter | Estimate | Standard Error | t Value | $\boldsymbol{P}_{\boldsymbol{r}}\mathbf{>}\left\vert\boldsymbol{t} \right\vert$ |
| LTN | 0.552482 | 0.428276 | 1.290 | 0.00414 |
| JOR | 0.14098 | 0.08314 | 1.696 | 0.22351 |
| PGDP | 0.006344 | 0.002020 | 3.141 | 0.11805 |
| Middle-level cluster | | | | |
| Parameter | Estimate | Standard Error | t Value | $\boldsymbol{P}_{\boldsymbol{r}}\mathbf{>}\left\vert\boldsymbol{t} \right\vert$ |
| LRS | $0.5940$ | $0.3450$ | 1.722 | 0.1234 |
| LTN | $0.62578$ | $0.3831$ | 1.633 | 0.1410 |
| LMS | $0.61853$ | $0.4532$ | 1.365 | 0.20949 |
| JOR | $0.28715$ | $0.1249$ | 2.298 | 0.0506 |
| PHF | $0.0002186$ | $0.000114$ | 1.922 | 0.0909 |
| DRW | $0.006781$ | $0.0002248$ | 3.017 | 0.01664 |
| Low-level cluster | | | | |
| Parameter | Estimate | Standard Error | t Value | $\boldsymbol{P}_{\boldsymbol{r}}\mathbf{>}\left\vert\boldsymbol{t} \right\vert$ |
| LTN | $1.02$ | $0.334$ | 3.059 | 0.0156 |
| LTI | $-1.18$ | 0.314 | -3.477 | 0.0083 |
| LPD | $0.696$ | 0.197 | 3.533 | 0.0077 |
| JOR | $0.139$ | 0.0064 | 2.134 | 0.0653 |
| PGDP | $0.013$ | 0.002 | 5.245 | 0.000778 |
| LDW | $1.541$ | 0.382 | 4.037 | 0.003750 |

**Appendix E**

Table S6. the Shapiro-Wilk normality test of data

| Shapiro-Wilk normality test | |
| --- | --- |
| data: Initial_data$UPR | data: Log_Sr_data$LPV |
| W = 0.9496, p-value = 0.04905 | W = 0.9748, p-value = 0.4265 |
| data: Initial_data$RSC | data: Log_Sr_data$LRS |
| W = 0.43822, p-value = 6.708e-12 | W = 0.9835, p-value = 0.7621 |
| data: Initial_data$TON | data: Log_Sr_data$LTU |
| W = 0.8024, p-value = 2.687e-06 | W = 0.98843, p-value = 0.9286 |
| data: Initial_data$TOI | data: Log_Sr_data$LTI |
| W = 0.70453, p-value = 3.289e-08 | W = 0.96711, p-value = 0.2268 |
| data: Initial_data$DCI | data: Log_Sr_data$ICI |
| W = 0.85113, p-value = 3.916e-05 | W = 0.97167, p-value = 0.3325 |
| data: Initial_data$JOR | data: Log_Sr_data$LMS |
| W = 0.93366, p-value = 0.01266 | W = 0.98225, p-value = 0.7116 |
| data: Initial_data$PHF | data: Log_Sr_data$LPD |
| W = 0.90026, p-value = 0.000965 | W = 0.9707, p-value = 0.307 |
| data: Initial_data$MIS | data: Log_Sr_data$LUP |
| W = 0.84465, p-value = 2.674e-05 | W = 0.96499, p-value = 0.1889 |
| data: Initial_data$ROA | data: Log_Sr_data$RJR |
| W = 0.97678, p-value = 0.4953 | W = 0.97524, p-value = 0.4414 |
| data: Initial_data$PGDP | data: Log_Sr_data$LPH |
| W = 0.92769, p-value = 0.007778 | W = 0.97342, p-value = 0.3828 |
| data: Initial_data$POD | data: Log_Sr_data$ROA |
| W = 0.63991, p-value = 2.916e-09 | W = 0.97678, p-value = 0.4953 |
| data: Initial_data$DRW | data: Log_Sr_data$DRW |
| W = 0.9693, p-value = 0.2731 | W = 0.9693, p-value = 0.2731 |
| data: Initial_data$PVL | data: Log_Sr_data$LPG |
| W = 0.80304, p-value = 2.775e-06 | W = 0.98239, p-value = 0.7172 |
